# Canine Pyothorax: Comparison of Culture and Susceptibility Results to the BSAVA PROTECT ME Poster and Other Published Recommended Antimicrobial Use Guidelines

**DOI:** 10.3390/ani13243843

**Published:** 2023-12-13

**Authors:** Iris Heinsoo, David J. Walker, Kine Bergum Hjellegjerde, Julia W. Y. Tang, Alison L. Moores

**Affiliations:** 1 Anderson Moores Veterinary Specialists, The Granary, Bunstead Barns, Poles Lane, Hursley, Winchester SO21 2LL, UK; 2 Lumbry Park Veterinary Specialists, CVS Group Plc, Selborne Road, Alton GU34 3HL, UK; 3 Dick White Referrals, Station Farm, London Road, Cambridge CB8 0UH, UK

**Keywords:** thoracic empyema, pleural effusion, antibiotics, antimicrobial resistance

## Abstract

**Simple Summary:**

Pyothorax, a collection of pus in the pleural cavity (space between the lung and the inner surface of the chest wall), can be caused by penetrating injuries, migrating plant material, ruptured lung abscess, the spread of infection from pneumonia, or by way of the bloodstream. Antibiotic treatment recommendations for dogs with pyothorax whilst pending bacterial culture results are reported in the literature. The aim of this study is to assess the appropriateness of the current antimicrobial treatment recommendations by comparing them with the pleural fluid bacterial culture and antimicrobial susceptibility results. Fifty-three dogs were included, with 57.7% having a positive pleural fluid culture. The most commonly isolated bacteria were *Pasteurella* species (23.3%), *Escherichia coli* (23.3%), and mixed anaerobes (20%). Our results show that the recommended combination of potentiated amoxicillin and marbofloxacin would have been appropriate for most dogs. However, there was a high rate of resistance to clindamycin, which is one of the antibiotics that is included in the current treatment recommendations. Therefore, this antibiotic should not be used alone and may be less effective in combination therapy compared to potentiated amoxicillin.

**Abstract:**

The most common bacterial isolates in dogs with pyothorax include mixed anaerobes, Enterobacteriaceae (especially *Escherichia coli*), *Pasteurella* spp., *Streptococcus* spp., and *Staphylococcus* spp. A fluoroquinolone with amoxicillin (±clavulanate) or a fluoroquinolone with clindamycin are the most commonly recommended empirical antimicrobials whilst pending bacterial culture of the pleural effusion. The aim of this study is to review and compare the pleural effusion culture and antimicrobial susceptibility results to the PROTECT ME poster and other published antimicrobial use guidelines. The medical records of 53 dogs diagnosed with pyothorax between 2014 and 2020 at two veterinary referral centres were reviewed. Information, including culture and susceptibility results, was assessed. Antimicrobial susceptibility panels varied; susceptibility to a particular antibiotic was calculated as a percentage of isolates tested against the same antibiotic. A total of 30 of 53 dogs (57.7%) had a positive pleural fluid culture. The most common isolates were *Pasteurella* species (23.3%), *Escherichia coli* (23.3%), and mixed anaerobes (20%). From the aerobic isolates, 73–83% were susceptible to a fluoroquinolone, 14/19 (74%) to amoxicillin, and 20/22 (91%) to potentiated amoxicillin. Resistance to clindamycin was documented in 9/13 (69%) aerobic isolates, with all Gram-negative bacteria (9/9) being resistant. The combination of potentiated amoxicillin with marbofloxacin would have been appropriate in most of the dogs (75–92.9%). This study shows a high rate of resistance to clindamycin, which is not a suitable option for monotherapy and may be less effective in combination therapy compared to potentiated amoxicillin.

## 1. Introduction

Pyothorax, a collection of purulent effusion within the pleural space, can be caused by multiple underlying conditions, including direct inoculation by penetrating injuries to the thoracic wall, airways (e.g., inhaled migrating plant material) or oesophagus; haematogenous or lymphatic spread leading to disseminated infection; rupture of a lung abscess or necrotic pulmonary mass; and extension from discospondylitis or pneumonia [1,2,3,4]. On many occasions, the route of infection remains unknown [1,3,5,6].

Mixed anaerobes such as *Peptostreptococcus* spp., *Bacteroides* spp., *Fusobacterium* spp., *Prevotella* spp., *Clostridium* spp., and *Propionibacterium acnes*, as well as Enterobacteriaceae (especially *E. coli*), *Pasteurella* spp., *Streptococcus* spp., *Staphylococcus* spp., *Actinomyces* spp., and *Nocardia* spp., are the most common bacterial isolates in dogs with pyothorax [1,5,6,7].

A guide to the responsible use of antibacterials, the PROTECT ME poster, was published and revised in 2018 by the British Small Animal Veterinary Association (BSAVA) and the Small Animal Medicine Society (SAMSoc). Based on these guidelines, a fluoroquinolone with amoxicillin or potentiated amoxicillin or a fluoroquinolone with clindamycin are recommended as empirical antimicrobials whilst pending bacterial culture results in dogs with pyothorax. The same combinations, enrofloxacin or marbofloxacin with a penicillin or clindamycin, have also been recommended by the ISCAID (International Society for Companion Animal Infectious Diseases) Antimicrobial Use Guidelines for Treatment of Respiratory Tract Disease in Dogs and Cats [8]. Ampicillin–sulbactam has been suggested to be adequate in most cats, and adding a fluoroquinolone should be considered in dogs given ampicillin–sulbactam [3]. It has also been stated that ampicillin or clindamycin alone may be sufficient, but the authors commented that some anaerobes are resistant to ampicillin and clindamycin, and some *Pasteurella* isolates are resistant to clindamycin [3]. In addition to national and international antimicrobial use guidelines, antibiograms could complement this data by providing more specific recommendations based on the region [9]. Antibiogram development may, however, be complicated by too few isolate numbers being available; based on Clinical and Laboratory Standards Institute (CLSI) guidelines, only species with testing data for ≥30 isolates should be included [10].

In people, empirical antimicrobial therapy for community-acquired empyema (pyothorax) includes a second- or third-generation cephalosporin with metronidazole or a parenteral aminopenicillin with beta-lactamase inhibitor, whilst for hospital-acquired or postprocedural empyema, antimicrobials with activity for methicillin-resistant *Staphylococcus aureus* and *Pseudomonas aeruginosa* (e.g., vancomycin and cefepime or vancomycin and piperacillin/tazobactam) are recommended. Anti-anaerobic antibiotics (e.g., metronidazole, potentiated amoxicillin, and clindamycin) are advised in all cases regardless of culture results, and their use has been associated with better outcomes in human patients [11,12,13].

The aim of this study is to review cases of canine pyothorax and compare the pleural effusion bacterial culture and susceptibility results to the published recommended antimicrobial use guidelines for evaluating the appropriateness of empiric antibiotic selection based on current recommendations in the literature.

## 2. Materials and Methods

Medical records for cases of canine pyothorax were obtained retrospectively through a search of the term ‘pyothorax’ from medical records and microbiology reports at Anderson Moores Veterinary Specialists (AMVS), UK, between March 2015 and August 2020, and Dick White Referrals (DWR), UK, between April 2014 and February 2019. The inclusion criteria were the diagnosis of pyothorax based on gross and cytologic examination of pleural effusion, positive pleural effusion culture result, or both. Pleural fluid cytology was defined as positive when consistent with a neutrophilic exudate (degenerate or non-degenerate neutrophils with or without phagocytosed bacteria—cocci, rods, or mixed). Infection was cytologically classified as mixed in the presence of rods and cocci or morphologically different rod-shaped bacteria. The European Committee on Antimicrobial Susceptibility Testing (EUCAST) breakpoint guidelines were followed for susceptibility interpretation, except for one case where Clinical and Laboratory Standards Institute (CLSI) breakpoint guidelines were followed. Antimicrobial susceptibility testing was performed on an automated analyser (VITEK^®^, bioMérieux UK Ltd., Basingstoke, UK; 65.4% of samples) or using Kirby–Bauer disk diffusion susceptibility testing (34.6% of samples).

The following information was obtained for each case where available: age, sex, breed, body weight, presenting clinical signs, antimicrobial treatment prior to diagnosis, pleural effusion cytology, aerobic and anaerobic bacterial culture, antimicrobial susceptibility and resistance results, treatment, and outcome. All available categorical and continuous data were collected, anonymised, and entered into a spreadsheet (Microsoft Excel^®^ 2021; version 2310). Given the descriptive nature of this study, descriptive statistics are provided for continuous variables as mean or median (range) and categorical variables as a percentage. Antimicrobial susceptibility panels varied; therefore, susceptibility to a particular antibiotic was calculated as a percentage of isolates tested against the same antibiotic.

The culture and susceptibility results were tabulated and compared to the BSAVA PROTECT ME poster, ISCAID Antimicrobial Use Guidelines for Treatment of Respiratory Tract Disease in Dogs and Cats [8], and Canine and Feline Infectious Diseases textbook [3] treatment guidelines to assess how many would have received an appropriate antibiotic if these recommendations had been followed. Dogs were divided into three groups depending on whether the treatment based on the guidelines would have been appropriate (susceptible to at least one of the antimicrobials), inappropriate (resistant to the antimicrobial(s) in question), or of unknown efficacy, the latter because the antimicrobial(s) in question was/were not included in the susceptibility profile.

## 3. Results

### 3.1. Study Population

A total of 53 dogs with pyothorax were identified from AMVS (between March 2015 and August 2020) and DWR (between April 2014 and February 2019).

The median age was 3 years (range of 11 months to 11 years), and the median body weight was 18.05 kg (range of 7.1–49.9 kg). There were 33 males (62.3%; 19 were neutered, and 14 were entire) and 20 females (37.7%; 8 were neutered, and 12 were entire). A total of 11 dog breeds were represented, the most common being English Springer Spaniel (20/53, 37.7%) and Cocker Spaniel (16/53, 30.2%), followed by Labrador Retriever (5/53, 9.4%) and mixed-breed dogs (4/53, 7.5%). One dog of each of the following breeds was reported (1/53, 1.9%): Clumber Spaniel, Greyhound, Dogue de Bordeaux, Boxer, German Shepherd Dog, Hungarian Vizsla, Bernese Mountain Dog, and Italian Spinone.

### 3.2. Clinical Signs

Clinical signs at presentation are summarised in Table 1. The most common presenting signs were lethargy/weakness, pyrexia, tachypnoea, hyporexia/inappetence, and dyspnoea.

### 3.3. Diagnosis of Pyothorax

The pleural effusion cytology was compatible with the diagnosis of pyothorax in all cases. Two dogs with a neutrophilic pleural exudate had a negative culture and no bacteria detected on cytological examination but had historical, imaging, and surgical findings consistent with the diagnosis of pyothorax; both dogs had received antimicrobials prior to investigations.

Pleural fluid culture was performed in 52 (98.1%) dogs; thirty of the 52 dogs (57.7%) had a positive bacterial culture result. A total of 31 bacterial isolates were documented from dogs with positive pleural exudate cultures (Table 2). The most common bacterial isolates were *Pasteurella* spp., *E. coli*, and mixed anaerobes.

### 3.4. Comparison between Pleural Effusion Cytology and Culture Results

**Figure 1:**
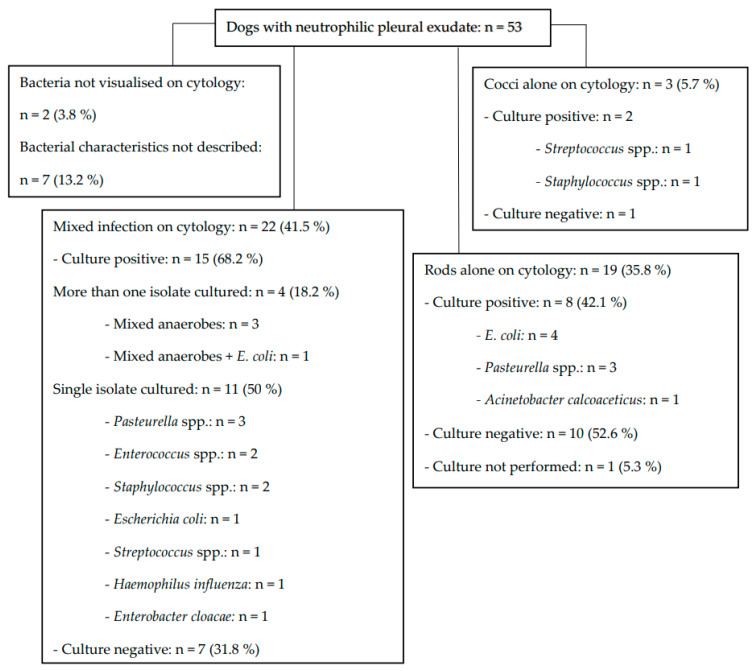
Cytology and culture results in canine pyothorax.

The pleural fluid cytology and culture results are summarised in Figure 1. Cytology was consistent with mixed infection in 22 dogs (41.5%); of these, more than one isolate was cultured in only 4 (18.2%) dogs. Regardless of the cytological findings, culture revealed at least two isolates in 6 (11.5%) dogs—mixed anaerobes (n = 5) and mixed anaerobes with *E. coli* (n = 1) were isolated.

### 3.5. Previous Antimicrobial Treatment

**Figure 2:**
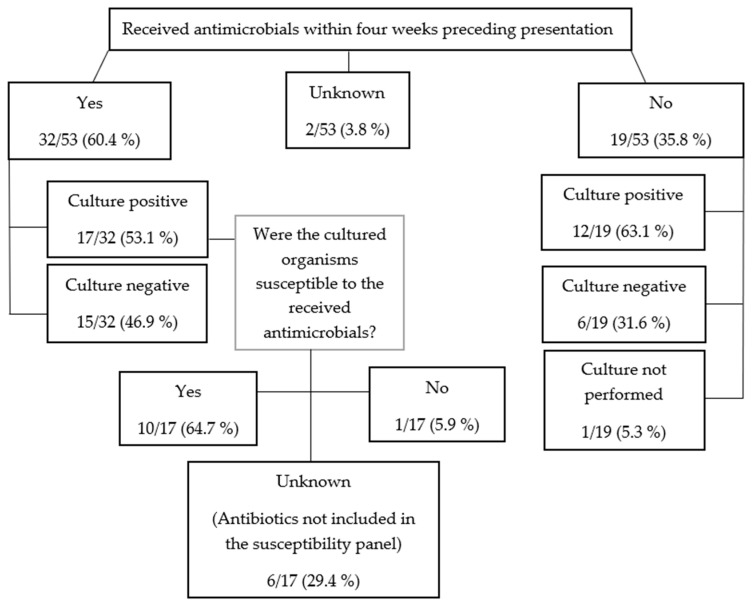
Previous antimicrobial treatment in canine pyothorax.

The previous antimicrobial treatment, along with whether the pleural effusion culture was positive or negative and whether the subsequently cultured organisms were susceptible or resistant to the initial choice of antibiotics, are summarised in Figure 2.

The most prescribed antimicrobial prior to referral was potentiated amoxicillin (19/32, 59.4%), followed by cefuroxime (3/32, 9.4%), and the combination of potentiated amoxicillin and marbofloxacin (3/32, 9.4%). The other antimicrobials or their combinations were each used in one (3.1%) dog and included amoxicillin, doxycycline, cephalexin, metronidazole, metronidazole with potentiated amoxicillin, enrofloxacin with potentiated amoxicillin, and a combination of enrofloxacin, cefuroxime, and potentiated amoxicillin. The mean duration of treatment prior to presentation was 2.5 days (range 1–10 days) for the dogs that were receiving antibiotics at the time of presentation (27/53, 50.9%). Two (2/53; 3.8%) dogs had received antibiotics within the past 4 weeks but had finished their course 2 days and 2 weeks before presentation. In three dogs (3/32, 9.4%), the length of previous treatment was unknown.

Only five of 32 (15.6%) dogs had received the appropriate combination of antibacterials based on the Antimicrobial Use Guidelines for Treatment of Respiratory Tract Disease in Dogs and Cats [8] and the BSAVA PROTECT ME poster (a fluoroquinolone with a penicillin or clindamycin), although one of the five dogs received an additional third antimicrobial (cefuroxime). Following referral, the proportion of dogs receiving the recommended empirical antimicrobials increased to 37.7% (20/53 dogs).

### 3.6. Pleural Effusion Culture and Susceptibility Results

The antimicrobial susceptibility of bacteria isolated from the pleural effusion in dogs is summarised in Table 3. The bacteria were divided into the following groups: all aerobes, which were subdivided into Gram-positive and Gram-negative isolates, and all obligate anaerobes. The data were presented in the following way due to the low numbers of each isolate (the recommended minimum of 30 isolates of each species to obtain a reliable percent susceptible statistic was not reached).

Antimicrobial susceptibility panels varied; only isolates tested against the antibiotic in question were used to calculate the percentage susceptible to the same antibiotic. From the aerobic isolates, 73–83% were susceptible to a fluoroquinolone (8/11 [73%] to enrofloxacin, 7/9 [78%] to pradofloxacin, and 19/23 [83%] to marbofloxacin). Thirteen aerobic isolates were tested only against marbofloxacin (11/13 susceptible, 2/13 resistant), eight isolates were tested against all three fluoroquinolones (6/8 susceptible, 2/8 resistant to marbofloxacin, enrofloxacin, and pradofloxacin), two were tested against marbofloxacin and enrofloxacin (1/2 susceptible to both, 1/2 susceptible to marbofloxacin but resistant to enrofloxacin), and one was tested against and was susceptible to both enrofloxacin and pradofloxacin. A total of 14 of 19 (74%) aerobic isolates were susceptible to amoxicillin and 20/22 (91%) to potentiated amoxicillin. More than 90% of the aerobes that were tested were susceptible to doxycycline (12/13), trimethoprim–sulfonamide (20/20), and gentamicin (10/10). Fewer than 65% of aerobic isolates tested were susceptible to cefoxitin (4/7), clindamycin (4/13), erythromycin (8/13), and fusidic acid (1/11).

Anaerobic isolates (mixed anaerobes) were only tested for and were all susceptible to metronidazole (5/5 [100%]).

No susceptibility panel was provided for one case with mixed anaerobic growth and for another case with *Streptococcus canis* growth; antimicrobials of choice were provided by the laboratory, but these were not included in Table 3 due to a lack of susceptibility testing.

Antimicrobials that were included in the susceptibility panel only on one or two occasions (e.g., cefovecin, cefpodoxime, vancomycin, and imipenem) were considered difficult to assess due to insufficient numbers.

Resistance to at least one antimicrobial was documented in 18 of 30 (60%) dogs with a positive pleural effusion culture.

### 3.7. Comparison of Bacterial Antibiotic Susceptibility to Antibiotic Guidelines

The susceptibility to the antimicrobials recommended for the treatment of pyothorax is summarised in Table 4. From the fluoroquinolones, marbofloxacin was chosen for comparison given that the susceptibility to enrofloxacin was lower (73%; see Table 3), and enrofloxacin was included in the susceptibility panel in fewer dogs compared to marbofloxacin (11 versus 23), which would have increased the proportion of dogs receiving treatment of unknown efficacy to as high as 60.7% (compared to 28.6% with marbofloxacin). Pradofloxacin was included in the susceptibility panel in only nine dogs and is not available in parenteral form. From the treatment guidelines in the Canine and Feline Infectious Diseases textbook [3], only clindamycin alone was used for comparison, as ampicillin was included in the susceptibility panel in only four dogs, likely due to its infrequent use compared to other penicillins in the United Kingdom. To assess the response to penicillin therapy alone, amoxicillin and potentiated amoxicillin were included in the comparison table instead of ampicillin.

A total of 2 of the 30 dogs with positive pleural effusion cultures were not included in Table 4, as no susceptibility profile was provided by the laboratory.

In five dogs, susceptibility to the recommended antimicrobials could not be assessed due to mixed anaerobic growth, which was only tested against metronidazole (including one dog with concurrent growth of *E. coli*). Additionally, susceptibility to marbofloxacin with amoxicillin could not be assessed in one dog with *Enterococcus* growth, which was resistant to marbofloxacin and was not tested against amoxicillin. Susceptibility to marbofloxacin with clindamycin could not be assessed in another three dogs—two dogs had a growth (*Enterococcus* spp. in the first and *Acinetobacter* spp. in the second dog) that was resistant to marbofloxacin and was not tested against clindamycin; one dog with *Pasteurella* spp. growth had neither marbofloxacin nor clindamycin in the susceptibility panel. Clindamycin was not included in the susceptibility panel for multiple infectious agents: mixed anaerobes (4), *Pasteurella* spp. (3), *E. coli* (2), *Enterococcus* spp. (2), *Enterobacter* (1), *Haemophilus influenza* (1), *Acinetobacter* spp. (1), and *Sphingomonas* spp. (1).

### 3.8. Treatment

In total, 25 of 53 (47.2%) dogs were treated surgically, and 28 of 53 (52.8%) dogs were managed medically, along with systemic antimicrobials. A total of 6 of 25 (24%) dogs that were treated surgically received appropriate empirical antimicrobials based on treatment guidelines (a fluoroquinolone with a penicillin or clindamycin), whilst 19/25 (76%) of dogs did not. Half of the dogs (14/28) treated medically received appropriate antimicrobials based on treatment guidelines, whilst half did not. Most of the medically treated dogs (24/28, 85.7%) were managed with thoracostomy tubes, whilst four (14.3%) dogs were treated with a single therapeutic thoracocentesis without lavage or repeated pleural effusion drainage.

### 3.9. Outcome

A total of 48 of 53 (90.6%) dogs survived to discharge (23/24 (95.8%) were managed with thoracostomy tubes, 23/25 (92%) underwent thoracotomy, and 2/4 (50%) were treated with therapeutic thoracocentesis), with a mean hospitalisation time of 7.5 days (range 2–20 days). Five (9.4%) dogs were euthanised (3) or died (2) during hospitalisation. The mean time to euthanasia or cardiopulmonary arrest in these 5 dogs was 2.2 days (range 0–9 days). One dog re-presented 35 days after discharge with marked bilateral pleural effusion and suffered cardiopulmonary arrest on the day of admission with unsuccessful resuscitation. Assessment of long-term outcomes was beyond the scope of this study.

A total of 19 of 20 (95%) dogs that received appropriate antimicrobials based on the treatment guidelines (a fluoroquinolone with a penicillin or clindamycin) survived to discharge, and one (5%) dog deteriorated and suffered cardiopulmonary arrest on the day of presentation. None of the dogs were managed with clindamycin alone. A total of 29 of 32 (90.6%) dogs that received inappropriate antimicrobials based on the treatment guidelines survived to discharge, whilst 3 (9.4%) died (1) or were euthanised (2). Both dogs were euthanised one day after admission due to the severity of the disease and possibly due to financial considerations—surgical treatment was discussed but declined in both cases. One dog that had not received antimicrobials was euthanised on the day of presentation due to a perceived guarded prognosis and recurring pyothorax 14 months after the first diagnosis and thoracotomy for lung lobectomy (at that time, *Nocardia* spp. infection was documented).

## 4. Discussion

Appropriate empirical antimicrobial therapy guidelines for dogs with pyothorax prior to the availability of susceptibility testing are essential for providing optimal treatment for the individual dog, but also on a larger scale by contributing to antimicrobial stewardship. This study shows that the commonly recommended combination of amoxicillin or potentiated amoxicillin with a fluoroquinolone for empirical treatment of pyothorax is appropriate in at least 71.4% and 75% of dogs, respectively, based on the culture results obtained. These combinations would have been inappropriate due to resistance in only 2/28 (7.1%) dogs.

Bacterial culture was positive in only 57.7% of dogs, which is comparable to earlier studies (55.5–92%) [6,7,14]. Our results show a discrepancy between pleural fluid cytology and bacterial culture results. Despite cytology showing a mixed bacterial infection in 41.5% of dogs, more than one bacterial isolate was cultured in only 11.5% of dogs. False negative cultures likely result from the combination of current/recent antimicrobial treatment and/or the presence of fastidious bacteria that are difficult to isolate. Our study is limited, as we are unable to assess whether antibiotic protocols suggested in the literature would be appropriate in those animals where there was a failure to culture any bacteria. Furthermore, it is possible that the effectiveness of proposed antibiotic protocols may have differed if all bacteria seen on cytological smears had been cultured from pleural fluid.

The susceptibility to amoxicillin with marbofloxacin and to potentiated amoxicillin with marbofloxacin could not be assessed in 6/28 (21.43%) and 5/28 (17.9%) dogs, respectively, because the antimicrobials in question were not included in the susceptibility profile. In five of those dogs, susceptibility could not be assessed due to mixed anaerobes being tested only against metronidazole. The antimicrobial activity of metronidazole and potentiated amoxicillin, graded from 0 to +++, has been reported to be excellent (+++) against beta-lactamase-producing and other anaerobes [15]. In one study, all obligate anaerobic isolates from different sites in dogs and cats were susceptible to potentiated amoxicillin, and most were susceptible to metronidazole [16]. Therefore, it is likely that the combination of potentiated amoxicillin with marbofloxacin would have been appropriate additionally in dogs where efficacy could not be assessed due to mixed anaerobes (17.9%), supporting the assumption that this combination could be appropriate in up to 92.9% of dogs.

Although amoxicillin alone can have excellent (+++) activity against some anaerobes, it lacks activity (0) against beta-lactamase-producing anaerobes [15], such as many *Bacteroides* spp. and some species of *Prevotella*, *Clostridium,* and *Fusobacterium* [17,18]. In our study, 74% and 91% of aerobic isolates in dogs were susceptible to amoxicillin and potentiated amoxicillin, respectively. Given the higher in vitro activity against both aerobic and anaerobic isolates, potentiated amoxicillin may be preferred over amoxicillin for the treatment of canine pyothorax. The drawback to using potentiated amoxicillin over amoxicillin is associated with the global concern over antimicrobial resistance. Based on the Categorisation of Antibiotics in the European Union (2020), Category C medication (potentiated amoxicillin) should be used when there is no clinically effective substance available in Category D (amoxicillin). Treatment with ampicillin alone could not be assessed as it was included in the susceptibility panel in only four isolates, but susceptibility to potentiated amoxicillin was nearly as high as to combination therapy with a fluoroquinolone, making it a potentially suitable option for monotherapy.

A total of 73% to 83% of aerobic isolates were susceptible to fluoroquinolones, with marbofloxacin showing the highest efficacy. Our results suggest that not using a fluoroquinolone with a penicillin (amoxicillin alone) would increase the proportion of dogs on inappropriate antibiotic therapy from 7.1% to as high as 21.4%. Based on the Categorisation of Antibiotics in the European Union (2020), treatment with Category B medication (all quinolones) should be restricted to when no lower-category antibiotics would be clinically effective. Fluoroquinolones, especially in earlier generations, have limited efficacy against anaerobic bacteria. Enrofloxacin has been reported to have almost no activity against anaerobic bacteria, and marbofloxacin only has a limited effect, while pradofloxacin has been reported to have enhanced activity against anaerobes compared to the other fluoroquinolones [8]. In one study, all anaerobic isolates were susceptible to pradofloxacin at 2 mg/L, whereas the MIC range extended to 64 mg/L for marbofloxacin and enrofloxacin [8,19]. In our study, mixed anaerobes were the third most common bacterial isolates, and the true incidence is likely to be higher as culturing these agents is challenging due to their complex metabolism and special (anaerobic) cultivation requirements [8,20,21]. Therefore, fluoroquinolones should only be used in combination with an antimicrobial with anti-anaerobic activity (e.g., potentiated amoxicillin, clindamycin, and metronidazole), regardless of culture results, due to possible fastidious anaerobic organisms. Whilst metronidazole has no activity (0) against aerobic bacteria and clindamycin is reported to be effective (+++) against Gram-positive aerobic cocci but not against Enterobacteriaceae (0), potentiated amoxicillin has good activity (++) also against aerobes [15]. Additionally, as clindamycin has been reported to have good (++) but reduced activity against anaerobes compared to metronidazole and potentiated amoxicillin (+++) [15], potentiated amoxicillin may be the most appropriate choice of empiric antimicrobial with anaerobic activity for the treatment of canine pyothorax.

Clindamycin is reported to have activity against most anaerobes and many Gram-positive bacteria but is known to be ineffective for most Gram-negative bacteria [8]. The treatment guidelines in the Canine and Feline Infectious Diseases textbook suggest clindamycin monotherapy as potentially effective for pyothorax, although they comment that some (not quantified) *Pasteurella* isolates are resistant [3]. A uniform resistance of *Pasteurella multocida* isolates to clindamycin has also been reported [22]. In our study, one of the highest rates of antibiotic resistance was to clindamycin (69% aerobic isolates), and all Gram-negative bacteria were resistant to clindamycin. Gram-negative bacteria accounted for 54.8% of bacterial isolates, with the most frequent isolates being Enterobacteriaceae (25.8%), and this is similar to previous studies [1,7,14]. These findings suggest that clindamycin monotherapy is not appropriate in dogs with pyothorax. Furthermore, given the high resistance rate of aerobes to clindamycin in our study, along with its previously documented reduced activity against anaerobes compared to potentiated amoxicillin, the combination of a fluoroquinolone with clindamycin is likely less effective compared to a fluoroquinolone with a penicillin.

An aminoglycoside was included in the susceptibility panel in 10 dogs, with all of the tested aerobic isolates being susceptible. However, obligate anaerobes are inherently resistant, aminoglycosides are potentially oto- and nephrotoxic, and they are not recommended as first-line antimicrobials as their use should be restricted for the treatment of multidrug-resistant organisms [8,23].

All aerobic isolates tested for trimethoprim–sulfonamide were susceptible; however, treatment with this antibiotic should be avoided due to possible inactivation of the drug by purulent material along with concerns regarding potential side effects such as blood dyscrasias, hepatopathy, and hypersensitivity reactions [3,8].

Some sources recommend discontinuation of fluoroquinolones, or either of the antimicrobials instituted empirically, if culture shows susceptibility to both [3,8]. However, given the discrepancy between our cytology and culture results, this approach may increase the risk of treatment failure in animals with polymicrobial infections undetected on culture. Therefore, if a positive clinical response to empirical treatment is seen and cytology suggests a polymicrobial infection when culture does not, continuing the first therapeutic regime unchanged might be warranted, but further studies would be needed to make firm recommendations.

The main limitations of the study include its retrospective nature, the difference in antimicrobials that were included in the susceptibility profiles, and the lack of quantitative data (minimum inhibitory concentrations) for antimicrobial susceptibility testing. The majority (60.4%) of dogs received antimicrobials prior to the collection of pleural fluid samples for bacterial culture, which may have affected the culture results. Larger multicentre studies would be required to reach a minimum of 30 isolates of each species for the development of regional respiratory antibiograms.

## 5. Conclusions

This study supports previous recommendations that a fluoroquinolone with a penicillin or clindamycin is an appropriate empirical antimicrobial choice for most dogs with pyothorax. The combination of potentiated amoxicillin with marbofloxacin may be the most appropriate treatment pending culture and susceptibility results. The rate of resistance to clindamycin is high, with all Gram-negative bacteria being resistant; therefore, clindamycin is not a suitable option for monotherapy and may be less effective in combination therapy compared to the penicillins.

## Figures and Tables

**Table 1:** Clinical signs in dogs with pyothorax; n = 53.

Clinical Signs	Dogs (%)
Lethargy/weakness	31 (58.5)
Pyrexia	30 (56.6)
Tachypnoea	25 (47.2)
Hyporexia/inappetence	22 (41.5)
Dyspnoea	20 (37.7)
Cough	12 (22.6)
Retching/vomiting/ hypersalivation	9 (17)
Panting	8 (15.1)
Weight loss	5 (9.4)
Perceived thoracic/abdominal pain	3 (5.7)
Diarrhoea	2 (3.8)
Lameness	1 (1.9)
Polyuria/polydipsia	1 (1.9)
Restlessness	1 (1.9)

**Table 2:** Bacteria isolated from the pleural effusion of dogs with pyothorax (%).

Aerobes		Obligate Anaerobes	Dogs (n = 30)
Gram-Positive	Gram-Negative		
	*Pasteurella* spp.		7 (23.3)
	*Escherichia coli*		7 (23.3)
		Mixed anaerobes	6 (20)
*Staphylococcus* spp.			3 (10)
*Streptococcus* spp.			2 (6.7)
*Enterococcus* spp.			2 (6.7)
	*Sphingomonas* spp.		1 (3.3)
	*Haemophilus influenza*		1 (3.3)
	*Enterobacter cloacae*		1 (3.3)
Total number of bacterial isolates			31

**Table 3:** Antimicrobial susceptibility of bacteria isolated from pleural effusion of dogs with pyothorax. Data presented as number susceptible/number tested, % in parentheses (rounded up or down to the closest full number).

Antimicrobial	All Aerobic Isolates; n = 24	Gram-Positive Isolates; n = 6	Gram-Negative Isolates; n = 18	All Anaerobic Isolates; n = 5
Ampicillin	3/4 (75)	2/2 (100)	1/2 (50)	
Amoxicillin	14/19 (74)	3/4 (75)	11/15 (73)	
Amoxiclav	20/22 (91)	5/5 (80)	15/17 (88)	
Cefalexin	17/20 (85)	3/4 (75)	14/16 (88)	
Cefotaxime	4/5 (80)		4/5 (80)	
Cefoxitin	4/7 (57)	1/2 (50)	3/5 (60)	
Ceftazidime	5/6 (83)		5/6 (83)	
Cefovecin	1/1 (100)		1/1 (100)	
Cefpodoxime	1/1 (100)		1/1 (100)	
Doxycycline	12/13 (92)	4/4 (100)	8/9 (89)	
Tetracycline	3/4 (75)		3/4 (75)	
Trimethoprim–sulfonamide	20/20 (100)	3/3 (100)	17/17 (100)	
Marbofloxacin	19/23 (83)	3/6 (50)	16/17 (94)	
Enrofloxacin	8/11 (73)	1/2 (50)	7/9 (78)	
Pradofloxacin	7/9 (78)	1/2 (50)	6/7 (86)	
Clindamycin	4/13 (31)	4/4 (100)	0/9 (0)	
Erythromycin	8/13 (62)	4/4 (100)	4/9 (44)	
Vancomycin	1/2 (50)	1/2 (50)		
Gentamicin	10/10 (100)	2/2 (100)	8/8 (100)	
Amikacin	3/3 (100)	1/1 (100)	2/2 (100)	
Chloramphenicol	2/2 (100)	1/1 (100)	1/1 (100)	
Metronidazole				5/5 (100)
Rifampicin	2/2 (100)	2/2 (100)		
Imipenem	2/2 (100)	1/1 (100)	1/1 (100)	
Nitrofurantoin	1/1 (100)	1/1 (100)		
Fusidic acid	1/11 (9)	1/2 (50)	0/9 (0)	

**Table 4:** Susceptibility to the published recommended antimicrobials for treatment of pyothorax in dogs (n = 28), % in parentheses.

Antimicrobials	Appropriate (Susceptible)	Inappropriate (Resistant)	Unknown Efficacy
Fluoroquinolone (marbofloxacin) + amoxicillin	20/28 (71.43)	2/28 (7.14)	6/28 (21.43)
Fluoroquinolone (marbofloxacin) + potentiated amoxicillin	21/28 (75)	2/28 (7.1)	5/28 (17.9)
Fluoroquinolone (marbofloxacin) + clindamycin	20/28 (71.4)	0/28 (0)	8/28 (28.6)
Clindamycin	4/28 (14.3)	9/28 (32.1)	15/28 (53.6)
Amoxicillin	14/28 (50)	6/28 (21.4)	8/28 (28.6)
Potentiated amoxicillin	19/28 (67.9)	3/28 (10.7)	6/28 (21.4)

## Data Availability

Data are contained within the article. The data presented in this study are available in the tables and figures in the article “Canine Pyothorax: comparison of culture and susceptibility results to the BSAVA PROTECT ME poster and other published recommended antimicrobial use guidelines”.
